# Sustainability-Driven Evaluation of Circular Plastic and Bioplastic Waste Reused as Building Materials Using MCDA and SWOT Analysis

**DOI:** 10.3390/polym18101176

**Published:** 2026-05-11

**Authors:** Maria-Paraskevi Belioka

**Affiliations:** 1 Lab of Polymer and Colours Chemistry and Technology, Department of Chemistry, Aristotle University of Thessaloniki, 54124 Thessaloniki, Greece; marivi.belioka@gmail.com or mpelioka@auth.gr; 2 Aachen Maastricht Institute for Biobased Materials, Faculty of Science and Engineering, Maastricht University, Brightlands Chemelot Campus, Urmonderbaan 22, 6167 RD Geleen, The Netherlands

**Keywords:** circular economy, (bio) plastic waste valorization, sustainable construction materials, material circularity, multi-criteria decision analysis (MCDA), SWOT analysis

## Abstract

The rapid accumulation of plastic waste has become a major environmental concern, while at the same time, it is necessary to create opportunities to rethink how these materials can be reintegrated into productive use, particularly within the construction sector. This study provides a sustainability-oriented review of the reuse of plastic waste, both fossil-based plastics and bioplastics, as building materials, with a specific emphasis on structured decision-support approaches. A systematic literature review was conducted to identify and analyze peer-reviewed studies examining the incorporation of plastic waste into construction applications, including composites, panels, insulation systems, and structural or non-structural components. Particular attention is given to research applying Multi-Criteria Decision Analysis (MCDA) and SWOT analysis as tools for evaluating sustainability performance across environmental, economic, technical, and social dimensions. The findings indicate that recycled plastic and bioplastic-based construction materials can deliver significant advantages, such as diverting waste from disposal pathways, reducing reliance on virgin resources, and, in certain cases, enhancing durability. However, these materials also face important challenges, including limitations in recyclability, concerns related to fire performance, regulatory acceptance, and uncertainties in end-of-life management. MCDA-based studies underscore the critical role of criteria selection and weighting, especially regarding environmental impact reduction and cost competitiveness, in shaping final rankings and decision outcomes. SWOT analyses, in turn, offer complementary strategic insights by highlighting issues related to market readiness, regulatory frameworks, and implementation barriers. By integrating these decision-oriented evaluation approaches, this review contributes to more transparent and evidence-based material selection processes and supports policy development aimed at strengthening circular economy strategies for plastic waste reuse in the built environment.

## 1. Introduction

Global plastic production has grown dramatically over the past two decades, reaching 460 Mt in 2019, while plastic waste generation rose from 156 Mt to 353 Mt over the same period. Despite sustained recycling efforts, only approximately 9% of plastic waste was ultimately recycled in 2019, with the majority either incinerated or landfilled [1,2]. This persistent imbalance between production and end-of-life management contributes to widespread pollution in terrestrial, freshwater, and marine ecosystems, as well as to greenhouse gas emissions throughout the plastics lifecycle [3,4].

Within this context, the construction sector represents both a major material sink and a significant opportunity for circular material use. Construction activities consume substantial quantities of raw materials and account for a considerable share of global resource extraction, energy use, and carbon emissions [5,6]. Although the sector handles large material flows, the reuse and recycling of plastic waste in building materials remains underdeveloped relative to its potential [4,6]. Applications such as panels, bricks, insulation systems, and composite materials demonstrate how plastic waste can be incorporated into construction products, thereby diverting waste from landfills and reducing reliance on virgin materials [7,8,9,10].

The key scientific gap this review addresses is the fragmented and inconsistent application of formal decision-support tools, and specifically Multi-Criteria Decision Analysis (MCDA) and SWOT analysis, in the evaluation of plastic and bioplastic waste reuse in construction [11]. While numerous studies examine technical performance and general sustainability advantages, comparatively few systematically analyze how sustainability decisions are structured, how criteria are selected and weighted, and how conclusions are justified across environmental, economic, technical, and social dimensions [12,13]. As a result, this limits the cross-study comparability and weakens the evidence base for informed policy and material selection decisions.

The main contribution of this review is therefore threefold: (i) to synthesize evaluation criteria, weighting approaches, and decision contexts from existing MCDA and SWOT-based studies on plastic waste in construction; (ii) to identify recurring patterns, methodological gaps, and underrepresented sustainability dimensions; and (iii) to propose an integrated MCDA–SWOT conceptual framework that links quantitative sustainability ranking with strategic feasibility analysis, supporting evidence-based material selection and circular construction policy.

### 1.1. Circular Economy and Plastics

A circular economy approach to plastics challenges the traditional linear “take–make–dispose” model by prioritizing the retention of material value through improved design, reuse, repair, and recycling. Circular economy frameworks seek to extend product lifecycles, recover valuable material resources, and close material loops while minimizing environmental impacts [14,15]. Despite increasing attention from industry and policymakers, progress toward circularity in plastics remains limited. As of 2022, only a relatively small share of global plastics production was classified as circular, and post-consumer recycled plastics accounted for a minor fraction of total material flows [16,17].

### 1.2. Scope and Material Classification

To ensure conceptual clarity, this review defines the scope of materials based on their origin and their potential for circular reuse in construction applications. Both fossil-based plastic waste and bioplastic waste are considered within a unified circular economy framework [18,19]. Rather than assuming bioplastics to be inherently sustainable, all material types are evaluated according to their performance and circular potential within the construction context [20,21,22].

Fossil-based plastics examined include polyethylene terephthalate (PET), high-density polyethylene (HDPE), low-density polyethylene (LDPE), polypropylene (PP), polyvinyl chloride (PVC), and polystyrene (PS). Bioplastics covered include polylactic acid (PLA), polyhydroxyalkanoates (PHAs), starch-based plastics, and bio-based PET (bio-PET). Both categories are evaluated through the same sustainability lens, emphasizing evidence-based assessment rather than feedstock-based assumptions [13,22,23,24,25,26].

### 1.3. Decision-Support Focus: MCDA and SWOT

Many traditional evaluations of plastic waste in construction focus on isolated indicators such as mechanical properties, cost, or selected environmental impacts, without adequately addressing the multidimensional nature of sustainability decision-making [11,12]. This review places explicit emphasis on MCDA and SWOT as structured decision-support tools that enable a more systematic evaluation of alternatives. Rather than simply classifying material innovations, this review examines how sustainability decisions are structured and how criteria are selected, weighted, and justified in the context of circular plastic and bioplastic waste reuse in construction.

## 2. Methodology

### 2.1. Systematic Review Framework and Purpose

A systematic literature review was conducted in accordance with the PRISMA (Preferred Reporting Items for Systematic Reviews and Meta-Analyses) guidelines to ensure transparency, reproducibility, and methodological rigor [27]. The purpose of this review is evaluative and synthesizing, as it aims to assess how sustainability is evaluated in studies on plastic and bioplastic waste reuse in construction, with a specific focus on structured decision-support tools (MCDA and SWOT). This review is not intended as a primary empirical study but as a critical synthesis of existing evidence to identify patterns, gaps, and best practices.

In terms of scope, this review is restricted to peer-reviewed journal articles published in English between 2010 and 2025, focusing on construction applications and sustainability assessment. The purpose of the review is to map the methodology by documenting which criteria are used, how they are weighted, and what conclusions are drawn. The intent is both academic (contributing to discourse on sustainability evaluation) and practical, offering guidance for material selection, policy design, and investment decisions in circular construction.

### 2.2. Literature Search Strategy and Database Selection

Searches were conducted in Scopus and Google Scholar. Web of Science was omitted from the final methodology as initial searches yielded results identical to those of the selected databases, particularly for emerging and interdisciplinary topics at the intersection of construction materials science, circular economy, and decision analysis. Scopus and Google Scholar collectively offer extensive coverage of engineering, environmental science, and sustainability journals, and were considered sufficient to capture the most relevant peer-reviewed literature within the defined scope. Backward and forward citation tracking of key methodological papers was also conducted to minimize the risk of overlooking relevant studies.

Search queries were developed by combining keywords across four thematic groups: (i) plastic waste and materials (plastic waste, bioplastic, recycled plastic); (ii) construction applications (building materials, construction, composites, insulation); (iii) sustainability concepts (sustainability, circular economy, lifecycle); and (iv) decision-support methods (multi-criteria decision analysis, MCDA, SWOT). A representative search string was (“plastic waste” OR “bioplastic waste” OR “recycled plastic”) AND (“building material” OR “construction”) AND (“sustainability” OR “circular economy”) AND (“MCDA” OR “multi-criteria decision” OR “SWOT”).

### 2.3. Inclusion and Exclusion Criteria

Studies were selected according to predefined criteria. Included studies were peer-reviewed journal articles focusing on the reuse of plastic or bioplastic waste in construction materials, with explicit consideration of sustainability aspects and application of MCDA methods and/or SWOT analysis (or structured multi-criteria evaluation). Excluded were studies focusing solely on mechanical or physical characterization without sustainability assessment, research addressing plastic waste incineration or energy recovery only, non-construction-related reuse pathways, and conference papers, editorials, or non-peer-reviewed sources.

### 2.4. Study Selection and Data Extraction

**Figure 1:**
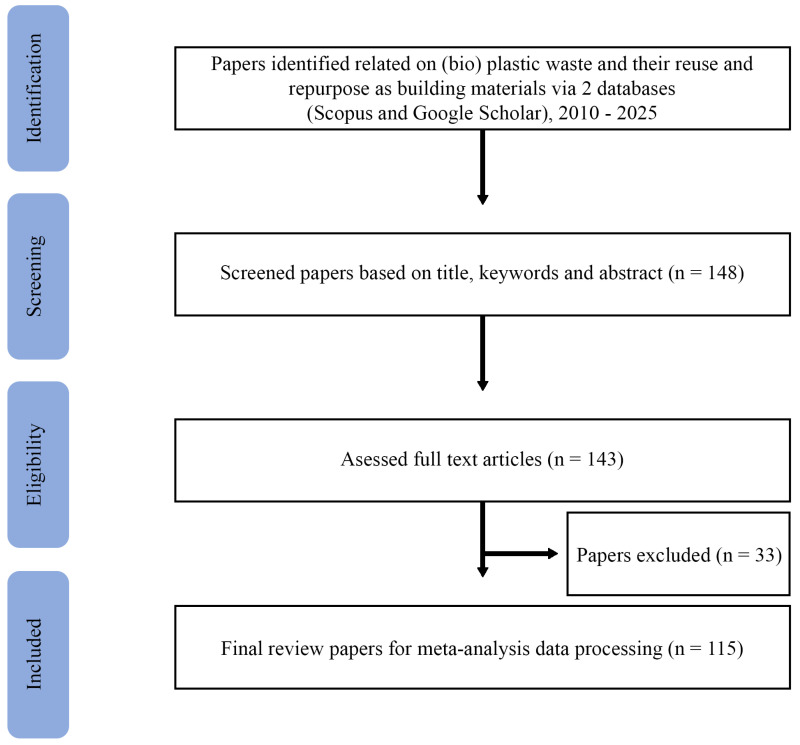
PRISMA literature review methodology.

After removing duplicates, titles and abstracts were screened for relevance. Studies that passed the initial screening underwent full-text evaluation to confirm alignment with the defined scope. For each selected study, data were extracted on material type, construction application, sustainability criteria considered, decision-support methods employed, and key findings. The extracted information was systematically coded to facilitate comparative analysis across application categories and evaluation frameworks. The overall selection workflow is illustrated in Figure 1 (PRISMA flow diagram).

## 3. Results and Discussion

### 3.1. Application Scope: Plastic and Bioplastic Waste in Construction

The reuse of plastic and bioplastic waste in construction materials has expanded considerably in recent decades, driven by growing environmental concerns, increasing regulatory pressure, and the broader adoption of circular economy principles [28,29]. To provide a structured and comparable sustainability assessment, this section classifies construction applications according to functional type rather than solely by polymer composition.

#### 3.1.1. Fossil-Based Plastic Waste

Fossil-based plastics account for the largest share of global plastic waste and represent the main feedstock for circular reuse in construction materials [29,30]. PET is widely used in panels, fibers, and composite materials; its tensile strength and chemical stability make it suitable for reinforcement and semi-structural applications [18,31]. HDPE and PP are often employed in lumber substitutes, decking systems, paving blocks, and molded bricks, valued for their moisture resistance and ease of processing. PVC presents a distinct case due to concerns related to plasticizers, stabilizers, and chlorine content, which can complicate recycling processes and raise regulatory concerns [24]. Polystyrene, particularly in expanded and extruded forms, is primarily reused in insulation and lightweight applications [6,8,32,33].

#### 3.1.2. Bioplastic Waste

PLA is the most extensively investigated bioplastic in construction-related applications, particularly in panels, lightweight boards, and blended composite systems. PHA and starch-based plastics have primarily been examined in experimental composite and hybrid systems. A key challenge in reusing bioplastic waste in construction lies in balancing biodegradability with long-term durability, as well as navigating competing end-of-life pathways (industrial composting, mechanical recycling, chemical recycling, and material reuse) [28,34,35].

#### 3.1.3. Construction Application Types

Four principal application categories are considered in this review:

*Structural applications* remain relatively limited due to the strict mechanical, safety, and regulatory requirements associated with load-bearing construction components. Recycled PET, HDPE, and PP are most frequently investigated, typically in hybrid configurations combined with steel, concrete, or fiber reinforcements [20,36,37,38,39].

*Non-structural building elements* including wall panels, façade components, paving blocks, bricks, roofing tiles, and modular blocks, represent the most established and widely implemented area for plastic waste reuse. These face fewer regulatory constraints, allowing greater flexibility in material formulation and product design [20,36,37,38,40].

*Insulation and lightweight materials* exploit the low density, thermal insulating properties, and moisture resistance of plastic waste. Recycled polystyrene, PET foams, and polyethylene-based materials are commonly used, while PLA-based systems have been investigated in lightweight composites [20,36,37,38].

*Composite and hybrid systems* combine plastic or bioplastic waste with mineral fillers, industrial by-products, or reinforcing fibers. Common formulations include plastic–sand bricks, polymer–fly ash composites, plastic–wood composites, and fiber-reinforced boards [20,36,37,38,40].

#### 3.1.4. Practical Applications and Real-World Examples

To illustrate the practical relevance of plastic waste reuse in construction, several documented implementations are worth highlighting. In Ghana, recycled mixed plastic waste has been processed into paving blocks and road construction elements, providing a cost-effective alternative to conventional asphalt in low-traffic areas [22,41]. In India and New Zealand, PET and HDPE waste from post-consumer packaging streams have been incorporated into compressed bricks and non-load-bearing wall panels, reducing both material cost and landfill burden [23,42,43]. Expanded polystyrene (EPS) waste from packaging has been widely reused in thermal insulation boards across European markets, with documented thermal conductivity values competitive with conventional mineral wool products [44]. In experimental and demonstration projects, PLA-based biocomposite panels have been developed and tested for interior applications, combining lightweight properties with reasonable acoustic insulation performance [44,45]. Waste plastic bottle-based construction has also been explored in informal settlement contexts, particularly in Rohingya displacement camps, where structural walls made from PET bottles filled with sand demonstrated feasibility in low-income, resource-constrained settings [46,47]. These examples span diverse geographic, economic, and application contexts, illustrating both the breadth of potential and the variability in performance outcomes that motivate the need for structured, decision-oriented sustainability evaluation [44,48].

### 3.2. Sustainability Dimensions and Evaluation Criteria

The sustainability assessment of plastic and bioplastic waste reuse in construction requires the systematic consideration of multiple, often competing, performance dimensions [49]. Existing studies rely on a diverse set of indicators and metrics, reflecting variations in application contexts, stakeholder priorities, and methodological approaches. To support a coherent synthesis, this section organizes the sustainability criteria identified in the literature into four core dimensions: environmental, economic, technical, and social/regulatory [50,51,52].

#### 3.2.1. Environmental Dimension

Environmental criteria form a central pillar of sustainability evaluations. Greenhouse gas emissions (expressed in CO_2_-eq) are among the most commonly reported indicators, encompassing emissions associated with waste collection and sorting, reprocessing, manufacturing, transportation, and end-of-life treatment [3,48,53]. Waste diversion indicators quantify plastic waste redirected from landfilling or incineration toward material reuse [48,54]. Toxicity indicators address the potential release of hazardous substances (plasticizers, flame retardants, stabilizers, heavy metals) during processing, use, and end-of-life stages [55,56]. End-of-life indicators evaluate recyclability, recoverability, or disposal pathways after service life, including closed-loop recycling, open-loop recycling, energy recovery, landfilling, and, for bioplastics, composting [57,58,59].

#### 3.2.2. Economic Dimension

Economic criteria address financial feasibility and market competitiveness. Material cost indicators include the price of plastic waste feedstock and preprocessing expenses (sorting, cleaning, additives) [60,61]. Processing costs encompass energy consumption, labor, equipment operation, and quality control during material conversion [60]. Lifecycle cost indicators expand assessment beyond initial production to include installation, operation, maintenance, repair, and replacement over the service life, which is particularly important for identifying trade-offs between higher upfront costs and long-term durability savings [62].

#### 3.2.3. Technical Dimension

Technical performance criteria assess functional suitability, reliability, and long-term durability. Mechanical properties, like compressive strength, tensile strength, flexural strength, and impact resistance, determine load-bearing capacity and deformation response [45,63,64]. Fire performance is also a major consideration for polymer-based construction materials, with compliance often treated as a threshold criterion in MCDA frameworks [65,66]. Durability criteria evaluate long-term resistance to UV radiation, temperature variations, moisture, and chemical agents. Moisture resistance and dimensional stability are also critical for outdoor or high-humidity environments [67,68,69,70].

#### 3.2.4. Social and Regulatory Dimension

Social and regulatory criteria encompass societal acceptance, institutional frameworks, and governance conditions [71]. At the same time, health-related criteria address potential release of VOCs, microplastics, or other hazardous substances during processing, installation, and use. Compliance with building codes, material standards, and certification schemes is a critical determinant of market entry. Market-related criteria reflect perceptions of quality, reliability, aesthetics, and environmental credibility among architects, engineers, contractors, and end-users [65,72,73].

#### 3.2.5. Linking Application Types to Sustainability Criteria

To facilitate a systematic and comparable synthesis, Table 1 aligns construction application categories with the dominant sustainability dimensions and criteria most frequently emphasized in decision-support assessments.

### 3.3. Material Properties, Performance Challenges, and Reintroduction Barriers

A critical aspect of reusing plastic waste in construction is understanding not only the potential benefits but also the challenges of reintroducing these materials in terms of their intrinsic properties, processing behavior, and compatibility with construction performance standards [93,94]. Table 2 provides a structured overview of key material types, their main performance characteristics, and the principal barriers to large-scale adoption [95].

### 3.4. Applications of Multi-Criteria Decision Analysis in Evaluating Plastic-Based Building Materials

Multi-Criteria Decision Analysis (MCDA) methods are increasingly used to support sustainability-oriented decision-making in the assessment of plastic and bioplastic waste reuse in construction materials. These approaches facilitate the structured integration of environmental, economic, technical, and social criteria, enabling stakeholders to consider trade-offs and prioritize alternatives in situations characterized by complex and sometimes conflicting objectives [12,103].

#### 3.4.1. Common MCDA Methods

A range of MCDA techniques has been applied to evaluate plastic-based building materials, reflecting differences in decision contexts, data availability, and analytical objectives. The most commonly used methods include the Analytic Hierarchy Process (AHP), the Technique for Order Preference by Similarity to Ideal Solution (TOPSIS), PROMETHEE, ELECTRE, MAVT, and hybrid LCA–MCDA frameworks [103].

AHP is the most frequently applied MCDA method in the reviewed studies. It organizes decision problems into a hierarchical structure and derives relative weights through pairwise comparisons based on expert judgment. Its structured approach and ease of application contribute to widespread use, though outcomes can be sensitive to subjective judgments [104,105].

TOPSIS ranks material alternatives according to their relative distance from an ideal and a negative-ideal solution. It is frequently combined with AHP-derived weights in integrated AHP–TOPSIS models and is valued for its computational simplicity and ability to process quantitative performance indicators [99,105].

Outranking approaches such as PROMETHEE and ELECTRE appear in studies involving more complex decision environments, particularly those characterized by qualitative or uncertain data. Hybrid frameworks integrating lifecycle assessment (LCA) with MCDA represent a growing trend, allowing lifecycle environmental impacts to be evaluated within a broader decision-making framework [48,106].

#### 3.4.2. Application Contexts

MCDA methods are applied within three primary decision-making contexts: material selection (the most common, evaluating alternative plastic-based building products or material formulations); technology and process comparison (evaluating recycling, processing, and manufacturing technologies); and policy and strategic prioritization (supporting waste management or circular economy scenario analysis and infrastructure planning) [107]. Please see Table 3.

#### 3.4.3. Synthesis of Key Findings

Environmental indicators and particularly greenhouse gas emissions, waste diversion potential, as well as resource efficiency, are consistently assigned high weights in most MCDA studies, often exerting a dominant influence on final rankings when LCA indicators are incorporated [48,108]. MCDA outcomes are highly sensitive to weighting schemes; even modest adjustments in the weights assigned to cost or technical performance can alter the preferred alternative, underscoring the importance of transparent weighting and meaningful stakeholder engagement [97]. Although plastic-based construction materials often demonstrate favorable environmental performance, elevated processing costs, variability in feedstock quality, and limited economies of scale frequently reduce cost competitiveness. Ranking patterns also vary systematically by application type: structural uses tend to weight technical performance and regulatory compliance more heavily, while non-structural and insulation applications typically emphasize environmental and cost-related indicators [11,106].

#### 3.4.4. Summary Table of MCDA Applications in Reviewed Studies

**Table 3:** Summary of representative MCDA-based studies on plastic and bioplastic waste in construction.

Material/Application	MCDA Method	Main Criteria	Main Outcome	Source
**Plastic waste** **management**	AHP/TOPSIS	Environmental, economic, social, technical	Highlights method diversity and need for transparent weighting across studies	[12,97,99,109]
**End-of-life** **alternatives for waste plastics**	Modified MAVT	Environmental, economic, social impacts	Decision outcome depends on multi-dimensional trade-offs across sustainability pillars	[12,97,104,106,107]
**Recycled-plastic paver blocks**	TOPSIS	Mechanical strength, water absorption, thermal resistance, cost	Identifies most suitable recycled-plastic composition based on performance trade-offs	[48,99,103,110]
**Waste plastics +** **agro-waste** **composites**	AHP/TOPSIS/VIKOR	Environmental benefit, technical performance, economic feasibility, end-of-life	Selects most suitable waste plastic type for agro-waste composites under integrated MCDM	[34,48,99,106]
**Insulation materials in buildings**	AHP/TOPSIS/VIKOR	Environmental (LCA), economic(LCC), technical, social/health	Highlights lack of standardization in criteria/weights and importance of LCA/LCC integration	[48,99,105,107]
**Sustainable concrete with waste PET bottle-cap aggregates**	AHP	Concrete performance, durability, sustainability, feasibility	PET cap aggregates can support sustainable concrete; MCDM used to select best alternative	[97,105,107,109]
**Building parts** **selection** **LCA + MCDM**	AHP	Environmental (LCA), technical, economic, social	Demonstrates how combining LCA with MCDM supports sustainable building-part selection	[48,106,109,111]
**Ecological paving stones from plastic + glass waste**	AHP	Mechanical strength, water absorption, material contribution	Identifies favorable constituent combinations; notes some pavers may not meet high-traffic standards	[103,104,105,107,109]

### 3.5. SWOT Analysis of Circular Plastic and Bioplastic-Based Building Materials

**Figure 2:**
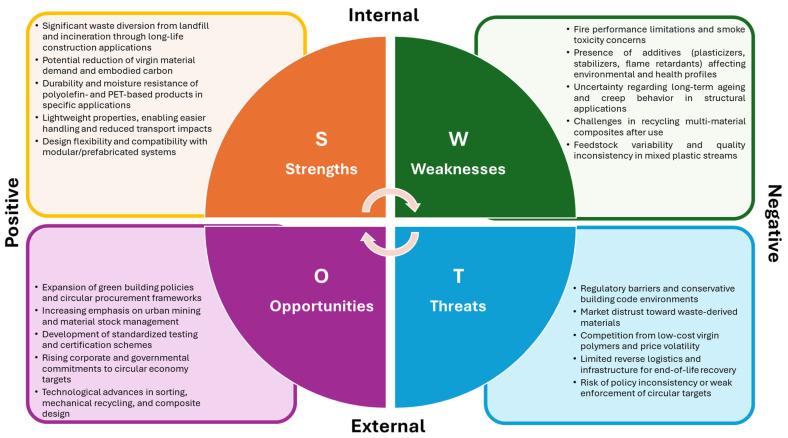
SWOT analysis for reusing plastic waste as a building material in the construction sector.

SWOT analysis is increasingly employed in the literature as a strategic evaluation tool to complement performance testing and sustainability assessments of recycled plastics in construction. From Figure 2 it is clear that unlike MCDA, which ranks alternatives based on defined criteria and weighting procedures, SWOT analysis contextualizes feasibility by identifying internal factors (strengths and weaknesses) and external factors (opportunities and threats) that influence real-world adoption, such as regulatory frameworks, market readiness, and supply-chain maturity [15,112].

#### 3.5.1. Strengths

*Waste reduction and diversion:* A consistently identified strength is the potential to divert substantial volumes of plastic waste from landfilling, incineration, and uncontrolled disposal by incorporating it into long-life construction products such as bricks, panels, and concrete components. By valorizing waste into durable building materials, these applications contribute directly to circular economy objectives [113].

*Durability and resistance in selected applications:* Recycled polyolefins (HDPE and PP) and PET-based components are frequently recognized for their resistance to moisture and chemical degradation, supporting suitability for outdoor environments and non-structural applications [114].

*Lightweight properties and functional flexibility:* Plastic-containing construction products generally exhibit lower density than conventional masonry units or mineral aggregates. This reduction in weight can improve handling and transportation efficiency and may facilitate modular or prefabricated construction systems [115].

#### 3.5.2. Weaknesses

*Fire behavior and smoke toxicity:* Fire performance remains a significant limitation for polymer-containing construction products. Even when materials demonstrate adequate mechanical strength or durability, fire-related performance constraints can restrict regulatory approval and limit broader adoption [71,116].

*Additives, toxicity, and leaching uncertainty:* The presence of plasticizers, stabilizers, and flame retardants can complicate recycling processes and raise concerns about emissions during manufacturing, installation, and use. These concerns are especially relevant for mixed or complex waste streams, including PVC fractions [24,114].

*Recyclability after use and multi-material challenges:* A recurring weakness is the difficulty of recycling plastic-based construction products at the end of their service life, particularly for composite and multi-material systems, where material separation is technically complex [113,115].

#### 3.5.3. Opportunities

*Green building policies and circular procurement:* Policy instruments such as recycled-content mandates, green public procurement schemes, and broader circular construction strategies create favorable conditions for scaling plastic-waste-derived building products [71].

*Urban mining and long-life material stocks:* The concept of buildings as long-term “material banks” presents an opportunity to integrate secondary plastics into durable construction applications, treating buildings as temporary repositories of valuable resources that may be recovered in the future [114].

*Standardization and certification development:* The development of harmonized product standards, performance testing protocols, and clear certification pathways represents a significant opportunity to reduce uncertainty and enhance market confidence in recycled plastic construction materials [89,113,115].

#### 3.5.4. Threats

*Regulatory barriers and conservative approval environments*: Even when laboratory-scale results demonstrate promising performance, adoption may be limited by conservative building code frameworks, the absence of harmonized technical guidance, and liability concerns [71].

*Market distrust and perception risks:* Perceptions associated with “waste-based” materials, regarding quality, safety, aesthetics, and long-term reliability, can act as barriers to wider acceptance [71].

*Competition with virgin materials and price volatility:* Fluctuations in virgin polymer prices, variability in feedstock quality, and the costs associated with sorting and processing can undermine the economic competitiveness of recycled plastic products [113,115].

#### 3.5.5. Why SWOT Complements but Does Not Replace MCDA

SWOT analysis is best understood as a strategic complement to MCDA rather than a substitute for it. MCDA structures trade-offs through clearly defined criteria, assigns weights reflecting stakeholder priorities, and produces transparent rankings of alternative options. SWOT provides contextual insight by explaining why an alternative that performs well in an MCDA framework may still encounter practical barriers, such as certification gaps, unfavorable public perception, or policy misalignment. Conversely, it can help identify conditions under which a lower-ranked alternative may become viable through procurement incentives, regulatory reforms, or evolving market standards. This complementarity is particularly important in circular construction, where successful adoption depends not only on environmental and technical performance but also on governance structures, supply-chain capacity, and market readiness [15,109,111,117].

### 3.6. Integrated MCDA-SWOT Framework for Decision-Oriented Circular Construction

Decision-making regarding the circular reuse of plastic and bioplastic waste in construction extends beyond technical feasibility or isolated sustainability indicators. Successful adoption depends both on systematically quantified trade-offs across environmental, economic, technical, and social dimensions, and on the broader contextual conditions that determine whether technically viable solutions can be implemented at scale [20].

Building on the insights developed in Section 3.2, Section 3.3, Section 3.4 and Section 3.5, this section proposes a conceptual MCDA–SWOT framework that integrates quantitative ranking methods with strategic feasibility analysis, providing a more comprehensive basis for material selection, policy development, and investment decisions within circular construction systems [109,117].

#### 3.6.1. Complementary Roles of MCDA and SWOT

MCDA enables explicit management of trade-offs, transparent prioritization aligned with stakeholder objectives, and sensitivity analysis to assess how rankings shift under different assumptions. SWOT captures contextual factors that are difficult to quantify but often decisive in real-world implementation, including market readiness, regulatory feasibility, supply-chain maturity, external policy drivers, and competitive pressures.

Rather than serving as alternatives to each other, MCDA and SWOT function in tandem: MCDA produces structured rankings, while SWOT explains the conditions under which those rankings translate (or not) into feasible implementation [109,117].

#### 3.6.2. How SWOT Can Inform MCDA Weighting

A central methodological contribution of this review is the formalization of how SWOT-derived insights can inform and strengthen MCDA weighting strategies. Based on the literature, four mechanisms are proposed [109,111,115,117]:

- *Constraint-driven weighting*: When SWOT highlights dominant threats such as fire safety concerns or regulatory barriers, critical criteria can be treated as non-compensatory threshold constraints, with alternatives required to meet predefined minimum standards before being included in the ranking.
- *Opportunity-aligned weighting:* When SWOT identifies strong enabling conditions such as green procurement policies or recycled-content incentives, MCDA weights can be aligned with these priorities by increasing emphasis on waste diversion, recycled content, and GHG reduction.
- *Risk-adjusted weighting:* If SWOT reveals supply-chain vulnerabilities, MCDA models can incorporate risk-adjusted weighting by assigning greater weight to feedstock quality stability, process robustness, and scalability.
- *Stakeholder-specific weighting profiles:* SWOT often reveals divergent stakeholder priorities, which MCDA can translate into multiple weighting profiles (regulatory, manufacturer, municipal, and client/market profiles).

#### 3.6.3. Proposed Conceptual Framework: Seven-Step Workflow

**Figure 3:**
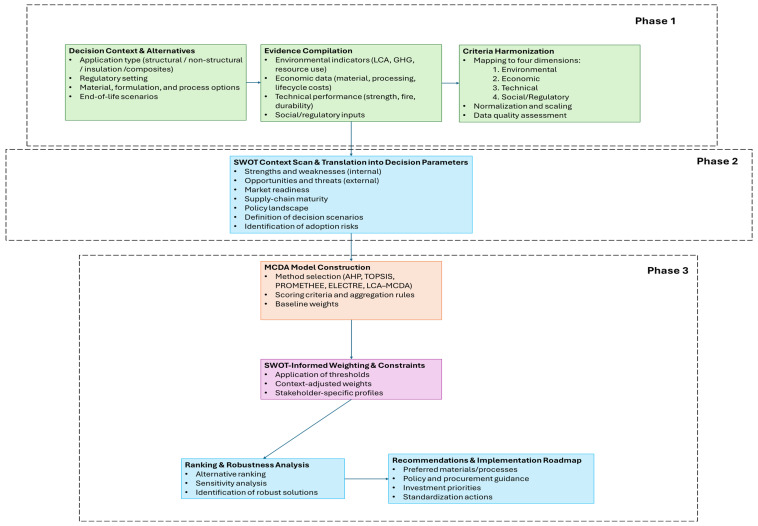
Phase 1 (Evidence and sustainability indicators), Phase 2 (context and feasibility assessment), and Phase 3 (decision modeling, implementation and outputs).

The proposed framework (Figure 3) links material performance data and sustainability indicators with MCDA-based prioritization and SWOT-based contextual analysis, ultimately producing context-aware and implementation-ready recommendations [109,111,115,117]. The workflow proceeds through seven steps:

- Step 1—Define decision context and alternatives: Specify application type, geographic and regulatory context, and alternatives under evaluation (polymer types, material formulations, processing routes, end-of-life strategies).
- Step 2—Evidence compilation and criteria harmonization: Map all performance indicators onto the four sustainability dimensions, ensure comparability through consistent normalization, and identify data gaps.
- Step 3—SWOT-driven context scan: Assess internal factors (strengths and weaknesses of each material) and external factors (opportunities and threats specific to the region, market, and regulatory environment).
- Step 4—MCDA model construction: Select the appropriate MCDA method (AHP, TOPSIS, PROMETHEE, VIKOR, MAVT, ELECTRE, or hybrid LCA–MCDA), define scoring rules and normalization procedures, and assign baseline weights.
- Step 5—SWOT-informed weighting and constraints: Introduce gating constraints, adjust weights to reflect dominant SWOT signals, and develop scenario-based weighting sets.
- Step 6—Ranking, sensitivity, and robustness analysis: Compute rankings under multiple weighting scenarios, conduct sensitivity analysis to identify potential ranking reversals, and identify robust alternatives.
- Step 7—Decision outputs and implementation roadmap: Translate results into final recommendations, implementation measures (standardization, certification, supply-chain improvements), and policy/investment guidance.

#### 3.6.4. Summary of Framework Insights: Key Decision Variables by Application Type

To support practical implementation of the proposed framework, Table 4 synthesizes the most decision-relevant variables across the four construction application types considered in this review. This summary is intended to assist practitioners in calibrating their MCDA models and contextualizing SWOT findings for specific application contexts.

### 3.7. Research Gaps and Future Directions

The reviewed literature highlights several persistent limitations that restrict methodological rigor, cross-study comparability, and practical relevance.

#### 3.7.1. Lack of Standardized Sustainability Criteria and Reporting Practices

The absence of standardized criteria sets and reporting protocols is the most significant limitation across existing MCDA and sustainability assessment studies. Many studies rely on customized indicators, normalization approaches, and scoring scales, which substantially limits cross-study comparability. Future research should focus on developing harmonized evaluation frameworks and establishing minimum reporting requirements, including clear documentation of data sources, system boundaries, weighting rationales, and sensitivity analyses [103,105].

#### 3.7.2. Underrepresentation of Bioplastic Waste in Construction Evaluations

Although bioplastics are becoming increasingly prevalent in consumer markets and waste streams, their reuse in construction materials remains underrepresented in sustainability assessments and MCDA-based research. The majority of existing evaluations focus on fossil-based polymers such as PET, HDPE, and PP. Future research should expand empirical evidence on bioplastic waste reuse, including field-scale applications and lifecycle-based assessments [97].

#### 3.7.3. Limited Integration of Social Lifecycle Assessment

Social dimensions remain inadequately addressed in most sustainability evaluations. Although environmental and economic indicators are frequently quantified, social impacts such as worker safety, community health, labor conditions within supply chains, and societal acceptance are often treated only qualitatively or omitted altogether. Future studies should incorporate social LCA methodologies to provide a more complete picture of sustainability performance [48,118].

#### 3.7.4. Uncertainty in End-of-Life Management

Many studies rely on simplified assumptions regarding disposal or recycling pathways, without adequately addressing uncertainties related to collection infrastructure, material degradation, contamination, and the economic feasibility of recovery. Future research should incorporate dynamic lifecycle modeling, scenario-based analysis, and material flow analysis. Greater emphasis on design-for-disassembly principles and mono-material strategies could significantly improve recyclability of plastic-based construction products [48,51].

#### 3.7.5. Regional and Geographic Bias

The reviewed literature reveals a clear regional imbalance, with most studies concentrated in Europe, East Asia, and a limited number of high-income countries. Future research should prioritize geographically diverse case studies and comparative analyses that reflect varied socio-economic and institutional contexts.

#### 3.7.6. Methodological Advancements for Integrated Decision-Support Frameworks

Future research should further develop hybrid MCDA–SWOT–LCA frameworks that incorporate probabilistic modeling, scenario-based weighting, and structured stakeholder participation. The use of digital decision-support tools and open-access databases could facilitate standardized data sharing and collaborative model refinement.

## 4. Conclusions

This review synthesized current knowledge on the reuse of fossil-based plastic and bioplastic waste in construction materials through a sustainability-driven and decision-oriented lens. The main findings can be summarized as follows:

- Plastic and bioplastic waste reuse in construction can meaningfully contribute to circular economy objectives by diverting waste from disposal pathways and embedding secondary materials in long-life applications. However, sustainability performance varies significantly across polymer types, construction applications, and regional contexts, and thus, it cannot be inferred from feedstock origin alone.
- MCDA provides valuable, quantitative decision support for managing sustainability trade-offs in material selection. Outcomes are, however, sensitive to criteria weighting, and greater standardization in criteria selection, reporting, and stakeholder engagement is needed to improve cross-study comparability.
- SWOT analysis offers a complementary strategic perspective that explains implementation feasibility. Regulatory frameworks, market acceptance, supply-chain maturity, and policy incentives are often as decisive as technical and environmental performance.
- The proposed integrated MCDA–SWOT framework addresses the gap between quantitative performance ranking and real-world implementation barriers. By linking structured ranking with strategic feasibility assessment, it enables more robust, context-sensitive, and actionable sustainability evaluations.
- Key priorities for future research include methodological harmonization, expanded assessment of bioplastics, integration of social lifecycle assessment, improved modeling of end-of-life scenarios, and geographically diverse empirical studies.

Advancing circularity in plastic waste management through construction applications requires not only technological development but also systematic decision science and coordinated governance. Integrated decision-support frameworks can play a pivotal role in accelerating the transition toward resilient, resource-efficient, and circular built environments.

## Figures and Tables

**Table 1:** Alignment between construction application types and dominant sustainability evaluation criteria in MCDA-based studies.

Application Type	Environmental Criteria	Economic Criteria	Technical Criteria	Social/Regulatory	Sources
**Structural** **Applications**	GHG emissions, embodied energy, resource efficiency	Lifecycle cost, maintenance cost	Mechanical strength, durability, creep, fire resistance	Code compliance, safety, liability	[3,8,22,30,38,74,75,76,77,78]
**Non-Structural Elements**	Waste diversion, material circularity, emissions	Material cost, manufacturing cost	Adequate strength, dimensional stability	Market acceptance, aesthetics	[8,14,28,30,36,52,79,80,81]
**Insulation and Lightweight**	Thermal performance, operational energy savings	Installation cost, energy cost savings	Thermal conductivity, moisture resistance	Fire safety, indoor air quality	[8,20,32,56,77,82,83,84,85,86,87]
**Composite and Hybrid** **Systems**	Multi-material impacts, recyclability	Processing complexity, scalability	Interfacial bonding, durability	Standardization, end-of-life management	[5,7,8,14,23,88,89,90,91,92]

**Table 2:** Overview of key plastic and bioplastic waste materials: properties, performance limitations, and barriers to reintroduction in construction applications.

Material	Key Properties in Construction	Performance Limitations	Barriers to Reintroduction	Sources
**Recycled PET**	High tensile strength, chemical stability, good fiber-forming ability	Moisture absorption, limited thermal resistance	Contamination in post-consumer streams; quality variability; additive uncertainty	[84,85,96,97,98]
**Recycled HDPE/PP**	Moisture resistance, durability, lightweight, processable	Creep under sustained load, poor fire performance	Limited structural application range; mixed waste streams reduce quality; fire code compliance challenges	[63,99,100,101,102]
**Recycled PVC**	Durability, weather resistance, wide availability	Additive complexity (plasticizers, stabilizers), chlorine content	Regulatory restrictions on certain additives; recycling process complexity; health concerns over VOC emissions	[24,63,99,100,101]
**Recycled EPS/PS**	Excellent thermal insulation, very low density	Flammability, poor structural contribution [63,99,100,101,102]	Strict fire safety regulations; low bulk density creates logistics challenges; styrene monomer concerns	[63,99,100,101,102]
**PLA**	Renewable origin, processable at low temperatures, good compatibility with natural fibers	Low thermal stability, brittleness, limited long-term durability outdoors	Competing end-of-life pathways (composting vs. recycling); limited industrial-scale construction applications; uncertain regulatory status	[28,34,58]
**PHA/Starch-based**	Fully bio-based, biodegradable, flexible formulations possible	High production cost, limited mechanical strength, moisture sensitivity	Very limited construction-scale evidence; high cost vs. conventional alternatives; supply chain immaturity	[28,34,58]

**Table 4:** Summary of key decision variables, MCDA recommendations, and implementation considerations by construction application type.

Application Type	Priority MCDA Criteria	Key SWOT Signals	Recommended MCDA Method	Implementation Considerations	Sources
**Structural**	Fire resistance, mechanical strength, regulatory compliance, lifecycle cost	Weakness:fire codes; threat: liability and certification gaps	AHP + TOPSIS with threshold constraints on fire/safety	Mandate fire and structural testing before ranking; engage certification bodies early	[105,109,111,115,117]
**Non-Structural Elements**	Waste diversion, cost-effectiveness, market acceptance, aesthetics	Strength: flexibility in design; Opportunity: green procurement	AHP or TOPSIS with economic and environmental criteria balanced	Prioritize low-cost, high-recycled-content formulations; engage architects and contractors	[99,105,111,115]
**Insulation and Lightweight**	Thermal conductivity, GHG savings, installation cost, fire behavior	Weakness: flammability; opportunity: energy efficiency regulations	LCA–MCDA hybrid to capture operational energy savings over full lifecycle	Balance thermal performance gains against fire safety compliance; target energy-efficiency schemes	[48,58]
**Composite and Hybrid Systems**	Recyclability, processing scalability, interfacial performance, end-of-life	Weakness: recyclability challenges; threat: market skepticism on multi-materials	PROMETHEE or ELECTRE for complex multi-attribute scenarios with qualitative criteria	Emphasize design-for-disassembly; develop clear end-of-life pathways before scaling up	[12,97,107,118]

## Data Availability

No new data were created or analyzed in this study. Data sharing is not applicable to this article.
